# Circulating mutational portrait of cancer: manifestation of aggressive clonal events in both early and late stages

**DOI:** 10.1186/s13045-017-0468-1

**Published:** 2017-05-04

**Authors:** Meng Yang, Umit Topaloglu, W. Jeffrey Petty, Matthew Pagni, Kristie L. Foley, Stefan C. Grant, Mac Robinson, Rhonda L. Bitting, Alexandra Thomas, Angela T. Alistar, Rodwige J. Desnoyers, Michael Goodman, Carol Albright, Mercedes Porosnicu, Mihaela Vatca, Shadi A. Qasem, Barry DeYoung, Ville Kytola, Matti Nykter, Kexin Chen, Edward A. Levine, Edgar D. Staren, Ralph B. D’Agostino, Robin M. Petro, William Blackstock, Bayard L. Powell, Edward Abraham, Boris Pasche, Wei Zhang

**Affiliations:** 1 Wake Forest Baptist Comprehensive Cancer Center, Wake Forest Baptist Medical Center, Medical Center Blvd., Winston-Salem, NC 27157 USA; 2 Department of Cancer Biology, Wake Forest School of Medicine, Winston-Salem, NC 27157 USA; 3 Department of Internal Medicine-Section of Hematology and Oncology, Wake Forest School of Medicine, Winston-Salem, NC 27157 USA; 4 Department of Biostatistical Sciences, Wake Forest School of Medicine, Winston-Salem, NC 27157 USA; 5 Department of Laboratory Medicine and Pathology, Wake Forest School of Medicine, Winston-Salem, NC 27157 USA; 6 Department of General Surgery-Section of Surgical Oncology, Wake Forest School of Medicine, Winston-Salem, NC 27157 USA; 7 Department of Radiology, Wake Forest School of Medicine, Winston-Salem, NC 27157 USA; 8 Department of Radiation Oncology, Wake Forest School of Medicine, Winston-Salem, NC 27157 USA; 9 Department of Social Sciences and Health Policy, Wake Forest School of Medicine, Winston-Salem, NC 27157 USA; 10 Institute for Biosciences and Medical Technology, University of Tampere, 33520 Tampere, Finland; 11 Department of Epidemiology and Biostatistics, Tianjin Medical University Cancer Institute and Hospital, 300060 Tianjin, China; 12 Wake Forest School of Medicine, Winston-Salem, NC 27157 USA; 13 Center for Genomics and Personalized Medicine Research, Wake Forest School of Medicine, Winston-Salem, NC 27157 USA; 14 Cancer Genomics and Precision Medicine, Wake Forest Baptist Comprehensive Cancer Center, Winston-Salem, NC 27157 USA

**Keywords:** Liquid biopsy, Non-invasive, Clonality, Mutation rate, Lung cancer

## Abstract

**Background:**

Solid tumors residing in tissues and organs leave footprints in circulation through circulating tumor cells (CTCs) and circulating tumor DNAs (ctDNA). Characterization of the ctDNA portraits and comparison with tumor DNA mutational portraits may reveal clinically actionable information on solid tumors that is traditionally achieved through more invasive approaches.

**Methods:**

We isolated ctDNAs from plasma of patients of 103 lung cancer and 74 other solid tumors of different tissue origins. Deep sequencing using the Guardant360 test was performed to identify mutations in 73 clinically actionable genes, and the results were associated with clinical characteristics of the patient. The mutation profiles of 37 lung cancer cases with paired ctDNA and tumor genomic DNA sequencing were used to evaluate clonal representation of tumor in circulation. Five lung cancer cases with longitudinal ctDNA sampling were monitored for cancer progression or response to treatments.

**Results:**

Mutations in *TP53*, *EGFR*, and *KRAS* genes are most prevalent in our cohort. Mutation rates of ctDNA are similar in early (I and II) and late stage (III and IV) cancers. Mutation in DNA repair genes *BRCA1*, *BRCA2*, and *ATM* are found in 18.1% (32/177) of cases. Patients with higher mutation rates had significantly higher mortality rates. Lung cancer of never smokers exhibited significantly higher ctDNA mutation rates as well as higher *EGFR* and *ERBB2* mutations than ever smokers. Comparative analysis of ctDNA and tumor DNA mutation data from the same patients showed that key driver mutations could be detected in plasma even when they were present at a minor clonal population in the tumor. Mutations of key genes found in the tumor tissue could remain in circulation even after frontline radiotherapy and chemotherapy suggesting these mutations represented resistance mechanisms. Longitudinal sampling of five lung cancer cases showed distinct changes in ctDNA mutation portraits that are consistent with cancer progression or response to *EGFR* drug treatment.

**Conclusions:**

This study demonstrates that ctDNA mutation rates in the key tumor-associated genes are clinical parameters relevant to smoking status and mortality. Mutations in ctDNA may serve as an early detection tool for cancer. This study quantitatively confirms the hypothesis that ctDNAs in circulation is the result of dissemination of aggressive tumor clones and survival of resistant clones. This study supports the use of ctDNA profiling as a less-invasive approach to monitor cancer progression and selection of appropriate drugs during cancer evolution.

**Electronic supplementary material:**

The online version of this article (doi:10.1186/s13045-017-0468-1) contains supplementary material, which is available to authorized users.

## Background

Tumors have been broadly classified as either hematopoietic or solid types. Hematopoietic cancers are derived from neoplastic cells of blood cell origin, and solid tumors are normally associated with a specific organ or tissue type. In the molecular and genomic era of medicine, there are two seemingly opposite trajectories in the characterization of cancer. One trajectory is the zoom-in approach, which divides each cancer type further into subtypes. In this effort, a traditionally recognized cancer type, such as lung cancer, can be further divided into multiple subtypes based on the molecular and genomic signature [1]. The opposing trajectory is the merging of multiple cancer types in the traditional arena into a common “molecular” cancer type. From this perspective, ovarian and prostate cancer cases can be more similar to each other rather than cases within their own organ-specific cancers [2–4]. The boundaries are also blurred between blood cancers and solid tumors with the recognition of CTCs in the blood of solid tumor cancer patients. CTCs have been emphasized as the transitory cells of a solid tumor leaving one organ and moving to another via the blood or lymphatic system resulting in metastatic disease. Advancement in detection technologies has increasingly shed light on the importance of CTCs in cancer diagnosis and prognosis [5–12].

It has long been recognized that tumor cells can release protein molecules into circulation, e.g., tumor markers such as PSA, CEA, and CA125 [13–15]. This, however, can occur without tumor cells entering circulation [16, 17]. The recognition that tumor cells do enter circulation led to the logical conclusion that all molecular events occurring in solid tumor cells might be reflected in circulation [18–22]. Indeed, mutations of tumor-related genes and epigenetic alterations in DNA methylation detected in tumor tissues have been detected in CTCs and cell-free plasma of the patients of all solid cancer types examined [23–25]. With the capacity of next generation sequencing significantly increasing in recent years, blood based “liquid biopsy” has gained recognition as a non-invasive approach to gain insight into the molecular and genomic-driver events during solid tumor progression, as a window to monitor cancer response to treatment, and as a means to guide targeted therapies based on detected actionable mutations [26–30].

There are, however, many unanswered questions regarding this emerging approach. First, will mutation rates detected in ctDNA be as valuable as mutation profiling in tumor tissues? Second, is there a specific mechanism to disseminate tumor tissue mutations into circulation? Third, would these metastasis-related events serve as more effective targets for intervention?

In this study, we evaluated the clinical value of assessing ctDNA using the Guardant360 platform in 177 patients with solid tumors, the majority of whom were treated for lung cancer. The results allow us to evaluate mutational portraits of solid tumors in circulation. We also present the initial success in application of this new genomics platform in cancer monitoring and treatment.

## Methods

### Patients and sample collection

We retrospectively reviewed the liquid biopsy results of 177 consecutive de-identified patients with diverse cancers who were seen at Wake Forest Baptist Comprehensive Cancer Center. Blood samples were collected between June 2015 and September 2016. Sociodemographic and clinical data including gender (male, female), age (<55, 55–65; 65.1–75; >75), body mass index (underweight, normal, overweight, obese), smoking history (current/recent, former, never), race (white, black, Asian, other), stage (I, II, III, IV, unknown), metastasis (0, 1, 2, 3+), tumor type, and vital status (alive, deceased) of each patient were collected. Smoking status was defined by self-reported smoking history obtained from Cancer Registry and/or Epic Electronic Medical Record. Never smokers were defined as respondents who smoked less than 100 cigarettes in their lifetime. Based on evidence that smoking cessation reduces cancer risk by half at 5 years, active smokers at the time of clinical data collection and those who had quit smoking within the previous 5 years were considered current/recent smokers [31]. We defined lung cancers as lung adenocarcinoma (LUAD), lung squamous carcinoma (LUSC), non-small cell lung cancer-not otherwise specified (NSCLC-NOS), and small cell lung cancer (SCLC), according to WHO classification. Overall survival was defined as the interval from the date of initial surgical resection to the date of last follow-up or death. Most of the statistical analysis was descriptive in nature. The sample size was determined by the available patients with genetic testing information. We also examined 37 lung cancer tissue samples with completed sequencing that were paired with blood samples. The study was approved by the local institutional review board, and all patients provided written informed consent for genetic analysis of their tumor and plasma samples prior to participation in this study.

### Extraction and quantification of ctDNA and tDNA

Blood samples (3–10 ml) were collected in EDTA tubes (BD Vacutainer, Beckton, Dickinson and Company) and centrifuged for 10 min at 1000 *g* within 4 h of the blood draw. The supernatant containing the plasma was further centrifuged at 14,000 *g* for 10 min at room temperature and was stored at −80 °C until analysis. Initially, the plasma samples were selected on the basis of their availability, and then, consecutively. DNA was extracted from plasma using the QIAamp DSP Virus Kit (Qiagen), according to the manufacturer’s instructions. A real-time quantitative PCR TaqMan Assay targeting GAPDH was used to measure plasma DNA concentration. tDNA was extracted from the fresh frozen biopsy sample using the AllPrep DNA/RNA Mini Kit (Qiagen) and was quantified with Qubit 2.0 (Life Technologies).

### Identification of genomic mutations by NGS

Next generation digital sequencing was performed using the Guardant360 test by Guardant Health., (Redwood City, CA; www.guardanthealth.com), a Clinical Laboratory Improvement Amendments (CLIA)-certified and College of American Pathologists (CAP)-accredited clinical laboratory (Guardant Health, Inc.). At the time of this study, this test identifies potential tumor-related genomic alterations via complete exon sequencing of 73 cancer-related genes in ctDNA extracted from plasma. ctDNA was extracted from plasma, and the amount of ctDNA was quantified using electrophoretic separation in a massively parallel capillary array system allowing for post-extraction high-throughput, high-resolution fragment size-specific data acquisition for each sample processed. The ctDNA was then analyzed by paired-end sequencing by synthesis utilizing an Illumina Hi-Seq 2000 platform and hg19 as the reference genome as described [32]. Digital sequences were reconstructed using Guardant Health’s proprietary bioinformatics algorithms, allowing the detection of 1–2 mutant fragments in 10 mL of blood with an analytic specificity greater than 99.9999%. Single nucleotide variants (SNV), variants of uncertain significance (VUS), amplification, deletion, and fusions were quantitatively reported.

Thirty-seven lung tissue samples were processed  by Foundation Medicine (Boston, MA) using the FoundationOne NGS panel. For the FoundationOne test, DNA was extracted from one or more 40-μm sections of FFPE tissue using the Maxwell 16 FFPE Plus LEV DNA Purification kit (Promega) and was quantified using a standardized PicoGreen fluorescence assay (Invitrogen). Library construction was performed using 50–200 ng of DNA sheared by sonication to approximately 100–400 bp before end-repair, dA addition and ligation of indexed, Illumina sequencing adaptors. Enrichment of target sequences was achieved by solution-based hybrid capture with custom biotinylated oligonucleotide bases. Enriched libraries were sequenced to an average median depth of >500× with 99% of bases covered >100× (IlluminaHiSeq 2000 platform using 49 × 49 paired-end reads) and were mapped to the reference human genome (hg19) using the Burrows-Wheeler Aligner and the publicly available SAM tools, Picard, and Genome Analysis Toolkit. Genomic alterations detected include base substitutions, insertions, deletions, copy number alterations, and selected gene fusions (http://foundationone.com/). Point mutations were identified by a Bayesian algorithm; short insertions and deletions determined by local assembly; gene copy number alterations identified by comparison to process-matched normal controls; and gene fusions/rearrangements determined by clustering chimeric reads mapped to targeted introns [33].

### Statistical analysis

Demographic information such as gender, age, BMI, smoking history, race, stage, metastasis, vital status, tumor type, as well as the dates of sample reception, dates of results, list of alterations, and drug(s) available were extracted from the medical reports and were analyzed. Non-synonymous somatic mutation calls were quantified. Patients were assigned to low or high mutation load groups based on the cohort median mutation number. We applied a Mann-Whitney-Wilcoxon rank sum [34] and a Kruskal-Wallis test [35] to compare mutation load with clinical variables with two groups and multiple groups, respectively. A Fisher’s exact test was used to determine association between smoking status (defined as an ordinal variable—current/recent, former, never smokers) and DNA damage genes. Overall survival was estimated using the Kaplan-Meier method. We evaluated the overall survival using the log-rank test. All significance tests were two-sided.

All lung cancer patients with available ctDNA and tDNA results were included in the main clonality analysis. Mutations called from ctDNA sequencing (plasma) were compared with mutations from tissue biopsy DNA sequencing (tumor). Tumor clonality was analyzed with R package “SciClone” [36]. Non-synonymous “short” mutations (i.e., missense, non-sense, frameshift, non-frameshift (in/del), promoter, and splice) were included in tDNA. Used VAF of these short mutations to get the multiple clones of each 37 paired patient, 26 patients had clonality analyze. Then we mapped the mutations of ctDNA to tDNA, labeled the same mutation genes as red. Analyses were performed using R version 3.3.0.

## Results

### Patient characteristics

The sample was comprised of 177 patients with diverse cancers who had a liquid biopsy next-generation sequencing ctDNA test performed on their plasma. Patients’ median age was 65 years old (range 26–90). Most were white (83.6%) with a history of smoking (current/recent: 29.4%; former >5 years: 45.8%). There was a predominance of advanced-stage (84.7%) versus early-stage (14.2%) cancers, and the most commonly represented cancers were lung adenocarcinoma (29.4%), non-small cell lung cancer (13.5%), and lung squamous carcinoma (11.9%) followed by head/neck cancer (9.0%), colorectal and cancer of unknown primary (CUP) (6.8%), pancreatic cancer (3.9%), other GI (3.4%), small cell lung cancer (3.4%), and other cancers. Table 1 provides the baseline patients characteristics.

**Table 1:** Demographics of patients

Characteristic	No. (%)
Gender	
Male	96 (54.2)
Female	81 (45.8)
Age	
< 55 years	38 (21.5)
55–65 years	55 (31.1)
65.1–75 years	57 (32.2)
75.1–90	27 (15.2)
BMI	
Underweight (<18.5)	12 (6.8)
Normal (18.5 < =BMI < 25)	74 (41.8)
Overweight (25 < =BMI < 30)	57 (32.2)
Obese (> = 30)	34 (19.2)
Smoking history	
Current/recent^a^	52 (29.4)
Former	81 (45.8)
Never	44 (24.8)
Race	
White or Caucasian	148 (83.6)
Black or African American	24 (13.6)
Asian	2 (1.1)
Other	3 (1.7)
Stage	
Stage I	13 (7.4)
Stage II	12 (6.8)
Stage III	28 (15.8)
Stage IV	122 (68.9)
Unknown	2 (1.1)
# of metastasis sites	
0	85 (48.0)
1	64 (36.2)
2	18 (10.2)
≥ 3	10 (5.6)
Vital status	
Alive	118 (66.7)
Dead	59 (33.3)
Tumor type	
Lung adenocarcinoma	52 (29.4)
Non-small cell lung cancer-not otherwise specified	24 (13.5)
Lung squamous carcinoma	21 (11.9)
Head/Neck	16 (9.0)
Colorectal	12 (6.8)
Cancer of unknown primary (CUP)	12 (6.8)
Other	9 (5.1)
Pancreas	7 (3.9)
Other GI	6 (3.4)
Small cell lung cancer	6 (3.4)
Breast	3 (1.7)
Kidney	3 (1.7)
Liver	3 (1.7)
Prostate	3 (1.7)

^a^Recent includes smokers who quit within the past 5 years

### Mutations in ctDNA and association with survival

**Fig. 1:**
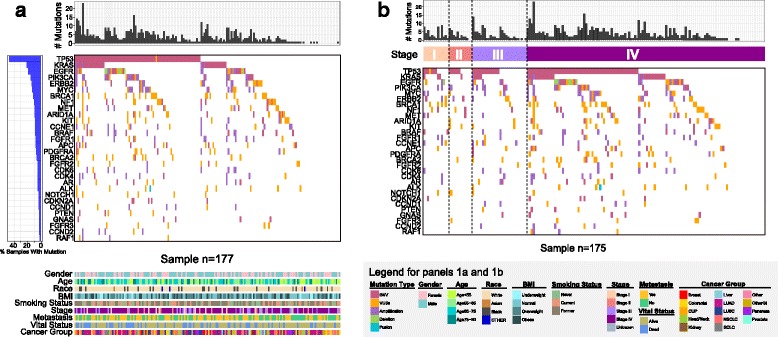
Global landscape of ctDNA mutations. **a** Global ctDNA mutational landscape of all patients for the top 30 genes having the largest fraction of mutations. *Top* and *left* bar charts show the number of mutations and percent of mutated samples, respectively. *Lower* part of panel A summarizes clinical information from each patient. **b** ctDNA mutational landscape of patients’ stage known for the top 30 genes having the largest fraction of mutations. Two patients’ stages are unknown

We detected 628 non-synonymous alterations in 61 of the 73 cancer-related genes from the Guardant360 ctDNA tests with a mean of 3.55 mutations per patient. Twelve genes were not found to be mutated in the whole sample. Eighteen patient plasma samples did not reveal mutations in the test (Additional file 1: Table S1). Figure 1a shows the top 30 most frequently mutated genes including *TP53*, *KRAS*, *EGFR*, *PIK3CA*, *ERBB2*, *MYC*, and *BRCA1*. The distribution of the top 30 mutated genes in the major cancer types is shown in Additional file 2: Figure S1.

We next examined whether the overall mutation rate in the plasma or any specific gene mutations were associated with clinical characteristics. The overall mutation rates in the plasma and the most commonly mutated genes (e.g., *TP53*, *EGFR*, *KRAS*) exhibited similar levels in early (I and II) and later (III and IV) stages (Fig. 1b). We performed a separate analysis for lung cancers (including LUAD, LUSC, NSCLC-NOS, and SCLC), which represented the majority of our cohort (103 of 172 cases) and found similar stage-independent mutation rates in the plasma (Additional file 2: Figure S2). We did not find any specific gene mutations associated with early stage cancers. In this particular cohort with a limited number of early stage cancers, we did not detect mutations in the following genes: *ARID1A*, *KIT*, *PDGFRA*, *BRCA2*, *FGFR2*, *ALK*, *CCND1*, *PTEN*, *GNAS*, *FGFR3*, *CCND2*, *and RAF1*.

**Fig. 2:**
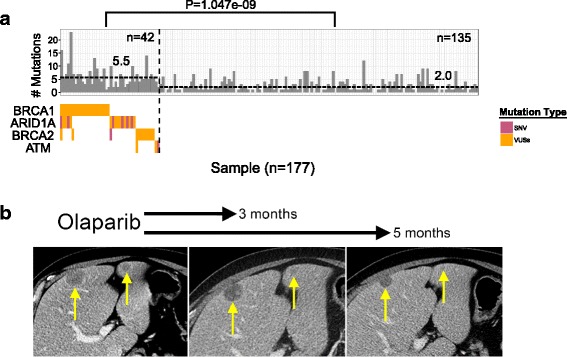
DNA damage repair (DDR) and chromatin remodeling gene mutations are associated with increased mutation number and may sensitize tumor to PARP inhibitor. **a** Patients with higher DDR and chromatin remodeling gene mutations numbers (*n* = 42) compared with patients with lower mutation numbers (*n* = 135). The *black dotted line* marks the median of the distribution. *** (*p* < 0.001), Mann-Whitney-Wilcoxon rank sum. **b** Composite image of axial contrast-enhanced computed tomography (CT) slicing through the liver demonstrates a progressive decrease in size of two hepatic metastases prior to treatment (*left*), 3 months (*middle*), and 5 months (*right*) after the initiation of olaparib therapy

The top-ranked mutated genes have been shown to be clinically valuable in designing targeted therapeutics. One group of genes that has gained attention is DNA ramage repair and chromatin remodeling genes. There was a significant difference in mutation load between patients with *BRCA1*, *ARID1A*, *BRCA2*, and *ATM* mutations and patients without these mutations (*p* = 1.047e^−09^, Mann-Whitney-Wilcoxon rank sum) (Fig. 2a). We also explored a patient diagnosed with prostate cancer who exhibited a *BRCA1* mutation in his ctDNA. The patient declined further cytotoxic chemotherapy; therefore, 28 months after his initial diagnosis of metastatic prostate cancer, he started receiving treatment with the PARP inhibitor olaparib. 3 months after initiation of olaparib, imaging revealed decrease in the size of multiple liver lesions, with the index lesion measuring 1.4 cm, from 2.9 cm prior (Fig. 2b). The patient responded well to olaparib for a total of 6 months before developing clinical evidence of disease progression.

**Fig. 3:**
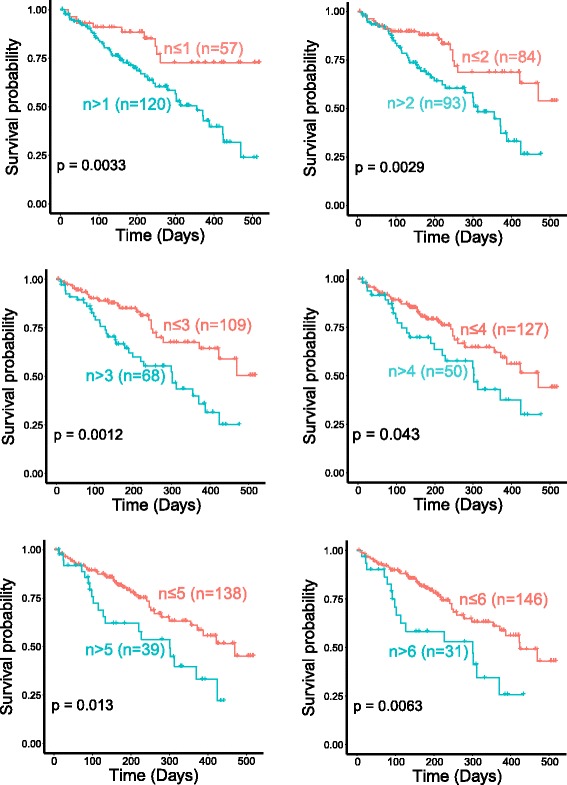
Higher mutation numbers in the ctDNA is associated with decreased survival. Higher mutation numbers in ctDNA is associated with poor survival. *n* defines the number of mutations, and survival plots are separated by mutation numbers: *n* = 1, 2, 3, 4, 5, and 6 mutations. *Blue lines* indicate more than *n* mutations, and the *pink lines* indicate equal to or less than *n* mutations. *P* values were derived using the log-rank test

A recent study reported that a mutation rate of three in the plasma using the Guardant360 platform was associated with survival in a pan-cancer study similar to ours [37]. We analyzed the relationship between mutation rates and survival in our cohort. We found that a mutation number of three did indeed significantly separate the patients into long and short survival groups. Further, we showed that this relationship between higher mutation rate and shorter survival was consistent using all cutoff values from one to six (Fig. 3).

We also found a slight association between mutation rates and age and BMI status in univariable (Mann-Whitney-Wilcoxon rank sum), but not in the multivariable test (Kruskal-Wallis) (Additional file 1: Table S2). The evaluation of mutation rate and the number of sites of metastasis showed that patients with more than three sites of metastasis had a significantly higher mutation rate than patients without metastasis (*p* = 0.02, Mann-Whitney-Wilcoxon rank sum). None of the 18 ctDNA mutation-free plasma samples, many from late stage cancer patients, had metastasized to more than two different distant organ sites (Additional file 1: Table S1).

### ctDNA mutations and clinical significance of smoking in lung cancer

**Fig. 4:**
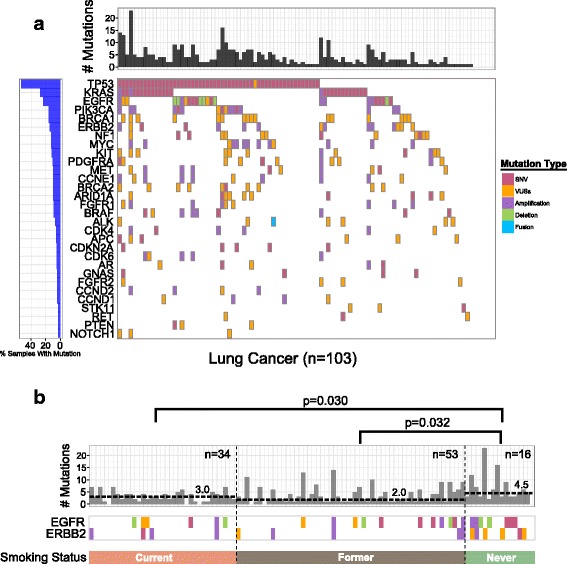
Gene mutations in lung carcinoma are associated with smoking status. **a** Mutational landscapes of lung cancers showing 30 of the most frequently mutated genes. *Top* and *left* bar charts show the number of mutations and percent of mutated samples, respectively. **b** *EGFR* and *ERBB2* gene mutations concentrate mainly in never smokers

WFBMC is located in Winston-Salem, NC at the epicenter of the US tobacco industry. As a consequence, smoking related cancers, especially lung cancers, are a critical public health issue in our catchment area, which consists of 58 counties surrounding Winston-Salem and extending into neighboring states. In our liquid biopsy project of Precision Oncology Initiative, 103 of the 177 patients were diagnosed with lung cancer, including adenocarcinoma (LUAD), squamous carcinoma (LUSC), non-small cell-not otherwise specified (NSCLC-NOS), and small cell lung cancer (SCLC) (Table 1). An analysis of gene mutations among the lung cancer subgroup showed top ranked mutations as *TP53*, *KRAS*, *EGFR*, and *PIK3CA* (Fig. 4a). There is a general consistency among the top ten mutated genes between Wake Forest patients and three [22, 37, 38] other published lung cancer cohorts (Additional file 1: Table S3). The notable difference is that *Myc* and *BRCA1* mutations are among the top ten mutated genes only in patients seen at Wake Forest.

There are 34 current/recent smokers and 53 former (>5 years) smokers, and only 16 never smokers among the lung cancer sample. We detect a higher mutation rate in never smokers than in current/recent and former smokers (Fig. 4b). Further, compared to current/recent (23.5%, 8/34) and former smokers (26.4%, 14/53), mutations in *EGFR* and *ERBB2* were significantly higher in never smokers (68.8%, 11/16; *EGFR*: *p* = 0.12, *ERBB2*: *p* = 0.02, *EGFR*, and *ERBB2*: *p* = 0.005, Fisher’s exact test).

Mutation number in the plasma is also correlated with survival in lung cancer cases: a higher number of mutations is associated with poorer survival (Additional file 2: Figure S3). Like the overall cohort, mutations in *TP53*, *EGFR*, and *KRAS* were detected in both early and late stages lung cancers (Additional file 2: Figure S2). Given these findings, one may hypothesize that the aggressive CTCs enter circulation at an early stage in lung cancer development when these cells likely represent a minor population of the whole tumor.

### Comparative analysis of the ctDNA and tDNA mutation results

**Fig. 5:**
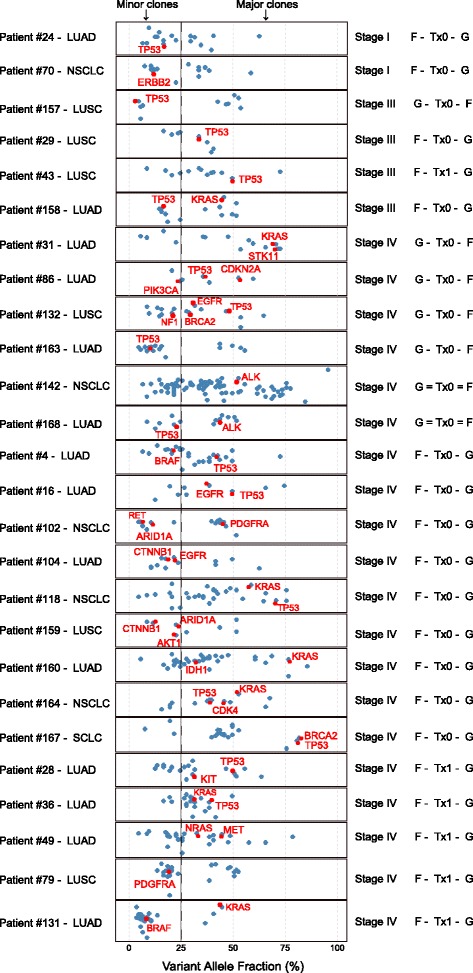
*TP53*, *PDGFBA*, *BRAF*, *ERBB2*, *CTNNB1*, *EGFR*, and *ARID1A* mutations present in minor clones in the primary tumors are detectable in plasma ctDNA. The X axis represents allele variant fractions. Each *circle* represents one gene mutation present in tumor tissues as examined by Foundation1 test (*F*). The cases presented manifest heterogeneity and multiple clonal characteristics. Mutations also found in Guardant360 test (*G*) are indicated by *red circle*. The order of the two tests and whether the patient was treated (*Tx1* for yes and *Tx0* for no) is shown in the right panel

To test whether CTCs as represented by the mutated genes enter circulation during early stage lung cancer, we performed detailed comparative analyses of mutation results in 37 lung cancer cases that had both tumor sequencing results by FoundationOne test and plasma sequencing result by Guardant360. The result of concordance test shows the percentage of top mutated genes in ctDNA and tDNA of these 37 lung cancer patients (Additional file 2: Figure S4). The two tests were performed on samples at different collection time except for two cases. Treatments were given for some cases between the two collection time points. We thus describe the cases based on the tumor stage and the sequence between FoundationOne test (F) and Guardant360 test (G) and whether there was treatment (Tx1) or no treatment in between (Tx0) (e.g., F-Tx1-G). The tumor heterogeneity is reflected by the variant allele fraction (VAF) based on F1 test in the X axis in Fig. 5. Among these 37 cases, 26 were successfully analyzed to present clonality endpoint.

In Fig. 5, each circle represents a gene mutation. The mutations also detected in plasma by Guardant360 test are indicated by red circles. For the stage I cases (*n* = 2), *TP53* and *ERBB2* mutations were found in a minor allele in the tumors with VAF less than 20%. Both mutations were detected in the plasma. For the stage III patients (*n* = 4), *TP53* and *KRAS* mutations were found in either minor (<25%) and major (>25%) populations in tumor tissues, and both could be detected in the plasma. For the stage IV patients (*n* = 20). Two cases had both tumor and blood collected in the same day. Both cases were positive for *ALK* fusion in both tests, and *ALK* fusions were present in the major population in the tumor. Four cases had blood drawn before surgery. Mutations of known driving mutations were found in minor populations in some cases, and these mutations (e.g., *TP53* in patient 157, *PIK3CA* in patient 86, *NF1* in patient 132, and *CTNNB1* in patients 104 and 159) were detected in plasma (Fig. 5). Similar patterns were seen in the cases where blood was drawn after surgery and in a number of the cases after the adjuvant chemotherapy or radiation therapy (Tx1). In two such cases, mutations of *PDGFRA* (in patient 79) and *BRAF* (in patient 131) found in the minor populations in the tumors were still present in the blood even after adjuvant therapy post-surgery suggesting they represent resistant clones (Fig. 5). These two patients could be candidates for targeted therapy using specific drugs for the mutated proteins.

### Monitoring of lung cancer progression by Guardant360 test

**Fig. 6:**
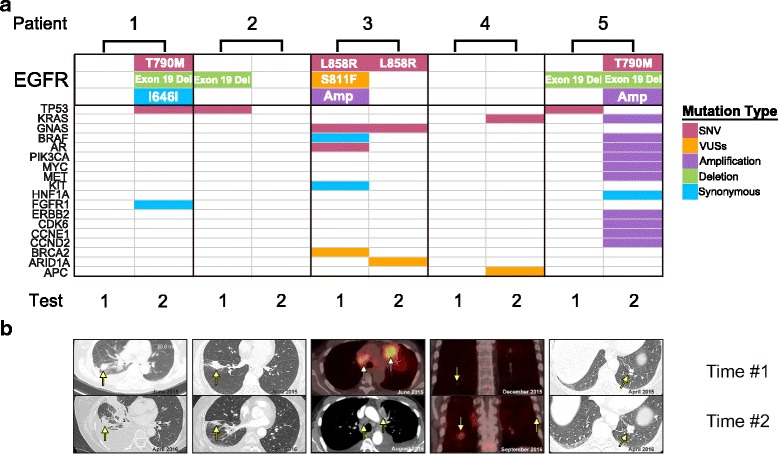
Monitoring of lung cancer progression and response to therapy through longitudinal plasma ctDNA sequencing. **a** Compared with ctDNA mutations in five patients at two different time points, *top* shows the patients’ number and relative time point. Four patients demonstrated *EGFR* mutations. Mutation burden of patients 2 and 3 decreased after treatment. **b** Serial imaging at the time of plasma ctDNA testing indicated partial response for patient 3, minor response for patient 2, and progressive disease for patients 1, 4, and 5

Mutations in *EGFR* represent one of the most common actionable targets for cancer treatment [39, 40]. A number of generations of inhibitors (e.g., erlotinib, gefitinib, afatinib, and osimertinib) have been developed to target specific EGFR mutations that are frequently found in untreated lung cancer and recurrent lung cancers [41–47]. Therefore, lung cancer management is known to benefit from longitudinal sampling, monitoring, and selection of different inhibitors [48]. The fact that *EGFR* mutations can vary over time further highlights the significance of longitudinal monitoring of multiple plasma samples during the disease progression and post-treatment. In our lung cancer patients, five patients were followed with this approach (patient no. 1–5 in Fig. 6).

Initially, the *EGFR* del 19 was detected in the original tissue biopsy of patient 1 based on PCR testing performed by our molecular pathology laboratory but had no *EGFR* del 19 mutation by Guardant360 testing (left panel for patient 1, Fig. 6). The patient received initial treatment with erlotinib and experienced a partial response with control of her disease for 7 months. The patient had disease progression, and the Guardant testing of the ctDNA at progression indicated the emergence EGFR T790M, and *EGFR* del 19. The patient was then treated with the T790M inhibitor, osimertinib, and experienced rapid clinical and radiographic improvement. Her initial follow-up CT scans indicated the presence of more sclerotic-appearing bone metastases. Her rapid clinical improvement after beginning osimertinib treatment corresponded to tumor flare in the recent CT scan image. Tumor flare is a clinical entity which is only observed in highly treatment-sensitive cancers. Subsequent imaging confirmed that these lesions represented flare, and she continued to respond to osimertinib for 6 months.

Patient 2 was successfully being treated with erlotinib and was found to have *EGFR* del 19 mutation and *TP53* mutation in the initial plasma sample. This patient eventually progressed on erlotinib and was treated by radiation and chemotherapy as well as immunotherapy. The second plasma sample was sent to investigate emergence of T790 during a period of progressive disease but had no detectable mutations by Guardant360 testing.

Patient 3 had EGFR L858R, S811F, and copy number amplification alterations in the first test. After treatment with erlotinib for 2 months, there was a significant decrease in tumor size shown in the CT scan. Plasma ctDNA test exhibited only one of the three *EGFR* alterations found before treatment.

Patient 4 did not have detectable ctDNA mutations by Guardant360 test during the initial evaluation for metastatic disease. This patient was treated by radiation and chemotherapeutic agents. 9 months later, the disease progressed with a significant increase in tumor size. Since the initial plasma sample was felt to be non-informative, the ctDNA testing was repeated and revealed emergence of *KRAS* and *APC* mutations.

Patient 5’s lung cancer had metastasized to the brain. *EGFR* Exon 19 del mutation was found in the initial plasma sample. She received gamma knife radiosurgery (GKRS) and started on erlotinib. She had stable systemic disease but experienced progression of CNS metastases despite erlotinib treatment. She received both GKRS as well as whole brain radiation therapy. She was judged to have meningeal carcinomatosis, and treatment was switched to pulse-dose erlotinib (1500 mg weekly) with concurrent pemetrexed. This provided some control of her disease for 10 months. She then experienced progressive neurologic decline and had Guardant360 testing, which revealed EGFR T790M mutation. After starting osimertinib, she experienced an improvement in her leg strength and concentration. She has been able to return to work part-time and continues to have control of her disease with osimertinib for 6 months.

## Discussion

The rapid advancement of deep sequencing has allowed development of clinical tests that sequence cell-free tumor DNA in circulation. Our understanding that solid tumor cells enter circulation or shed DNA into circulation created a pathway for a non-invasive approach to detect cancer and to monitor cancer progression over time. This approach is clinically valuable because tumor biopsy is often not possible or at least not feasible for multiple sampling during disease progression and management. As a part of the Precision Oncology Initiative at the Wake Forest Baptist Comprehensive Cancer Center in partnership with Guardant Health, we have processed plasma ctDNAs from 177 patients in several solid tumor types, the majority of whom have lung cancer. Our patient population is enriched with ever smokers because of the unique geographical and historical reasons that Winston-Salem, North Carolina serves as a major headquarters for tobacco manufacturing.

Analysis of the sequencing results has revealed several important insights. First, we found that the ctDNA mutation patterns are independent of tumor stage, regardless of whether we examined the total population of solid tumor patients or a subsample of lung cancer patients. This suggests that circulating tumor cells may enter circulation during an early stage of cancer progression, and the dissemination of tumor DNA is an early event. The best known tumor-associated mutations such as mutations in *TP53*, *KRAS*, and *EGFR* are found in both early and late stages of cancer. This is highly clinically significant and suggests that ctDNA mutation detection can be a non-invasive early detection approach that could potentially be applied to screening of high risk populations.

Second, mutation rates in ctDNA are a robust predictor of survival, which have not previously been reported using tumor tissues. This suggests that the ctDNA mutations, which may reflect a transition from local to systemic disease, represent a key clinical transformation. In other words, the ability of tumor cells to enter circulation may determine the disease outcome.

The finding of tumor stage-independent mutation patterns in plasma led us to hypothesize that the key for tumor heterogeneity investigations is to determine the clinically aggressive clones or clones that are resistant to therapies. To test this hypothesis, we have identified the lung cancer cases that had both tumor tissue DNA mutation detection using FoundationOne test and Guardant360 liquid biopsy test. More than 400 genes are included in FoundationOne test, clonality analysis can be carried out to exhibit clonalities based on allele frequencies of the mutated genes. Although such analysis is not feasible with Guardant360 test due to the lower mutation rate in general, we could match the mutations in plasma to these found in the tumor tissues. The results of our analysis showed that known mutations like *TP53* mutations are frequently found in plasma even when the mutations were found in a minor clone in the tumor tissues. Other gene mutations with similar behavior include *EGFR*, *BRAF*, *CTNNB1*, *ARID1A*, *ERBB2*, and *PDGFRA*. The results demonstrate that mutations in these genes are likely driver for metastasis through dissemination into circulation from circulating tumor cells.

Detailed examination of gene mutations in the cases where plasma ctDNA test was obtained after surgery and tumor DNA sequencing showed a large fraction of which had been treated by chemotherapy and radiation therapy. Nevertheless, mutations in genes like *BRCA1*, *BRAF*, and *PDGFBA* were still detected in the plasma suggesting that tumor cells harboring these mutations were not eliminated by the therapy and that these mutations may actually confer a resistance mechanism. An intriguing observation is that *BRCA2* mutations are more commonly found in tumor tissue exome sequencing but rarely in the plasma ctDNA mutation detection. This is consistent with the notion that *BRCA2* is a key double strand DNA repair gene product and mutations of which render the tumor cells sensitive to DNA damaging chemotherapy and radiation treatment [49, 50]. Although *BRCA1* is also involved in DNA repair, *BRCA1* is involved in a broader scope of cellular functions and mutations of which have been suggested to contribute to metastasis. These results suggest that comparative analysis of tumor tissue DNA mutation detection and plasma ctDNA mutation results can reveal mutational events that may sensitize or resist chemotherapy and radiation therapy. These mutational events may be better suited for targeted therapy. Given that, there are specific drugs that target *BRAF* [51–53], *PDGFRA* [54–56], and *ARID1A* [57] mutants, patients harboring these mutations could benefit from these specific treatments.

The emergence of clinical resistance to previously effective anti-neoplastic therapy results from the acquisition of molecular alterations in genes or pathways that govern resistance mechanisms. Defining these mechanisms of resistance to targeted agents is challenging because it is difficult to acquire serial tumor biopsies in patients with advanced disease. Therefore, we performed serial plasma ctDNA collections at two time points to detect the emergence of genomic alterations and acquired resistance to targeted therapy. At present, the most widely known tyrosine kinase inhibitor (TKI) targets in NSCLC-NOS are *EGFR* activating mutations, 90% of which consist of in-frame deletions in exon 19 and the L858R point mutation in exon 21. First-line treatment of patients harboring *EGFR*-activating mutations with *EGFR* TKIs gefitinib, erlotinib, or afatinib has resulted in superior overall response rates, progression-free survival, and quality of life compared to chemotherapy [58–60]. The acquisition of the T790M mutation is the most frequent resistance mechanism, responsible for nearly 60% of cases [1]. Several studies have reported the reduction or even disappearance during TKI treatment of *EGFR*-activating mutations in plasma, which had reappeared together with the T790M resistance mutation. Our study has validated the reports by others. We join investigators from other studies to propose the routine use of the longitudinal ctDNA testing as an effective way to monitor cancer progression, which can potentially lead to real-time changes in treatment.

## Conclusions

In summary, our liquid biopsy project provides supporting evidence that ctDNA mutation detection constitutes less-invasive real-time surrogates for early diagnosis, prognosis, therapeutic tailoring, and resistance monitoring and mitigates needle biopsy sampling errors related to intra- or inter-tumor heterogeneity.

## Additional files

Additional file 1: Table S1. Clinical and treatment characteristics of the 18 cases with no ctDNA alterations detected. **Table S2**. Description of mutation load in total cohort (*n* = 177). **Table S3**. All mutations detected in patient ctDNA compared with other studies. (DOC 625 kb)

Additional file 2: Figure S1. ctDNA mutational landscape for major cancer groups for the top 30 genes in total cohort. **Figure S2**. ctDNA mutational landscape of patients’ stage known for the top 30 genes in lung cancer cohort. **Figure S3**. Higher mutation numbers in the ctDNA are associated with decreased survival in lung cancers. Higher mutation numbers in ctDNA are associated with poor survival. “*n*” defines the number of mutations and survival plots are separated by mutation numbers: *n* = 1, 2, 3, 4, 5, and 6 mutations. Blue lines indicate more than “*n*” mutations and the pink lines indicate equal to or less than “*n*” mutations. P-values were derived using the log-rank test. **Figure S4**. The concordance of top mutated genes in the ctDNA and tDNA of 37 lung cancer patients. (DOC 1098 kb)

## Abbreviations

CTCs: Circulating tumor cells
ctDNA: Circulating tumor DNAs
CUP: Cancer of unknown primary
GKRS: Gamma knife radiosurgery
LUAD: Lung adenocarcinoma
LUSC: Lung squamous carcinoma
NSCLC-NOS: Non-small cell lung cancer-not otherwise specified
SCLC: Small cell lung cancer
SNV: Single-nucleotide variants
TKI: Tyrosine kinase inhibitor
VAF: Variant allele fraction
VUS: Variants of uncertain significance
