# Supplementary material for: Circulating mutational portrait of cancer: manifestation of aggressive clonal events in both early and late stages
Source: J Hematol Oncol. 2017 May 4;10:100. doi: 10.1186/s13045-017-0468-1 (PMC5418716; doi:10.1186/s13045-017-0468-1)
Supplement: Supplementary file 1 — Clinical and treatment characteristics of the 18 cases with no ctDNA alterations detected. Table S2. Description of mutation load in total cohort (n = 177). Table S3. All mutations detected in patient ctDNA compared with other studies. (DOC 625 kb) [file 13045_2017_468_MOESM1_ESM.doc]

**Additional file 1:**

| Table S1. Clinical and treatment characteristics of the 18 cases with no ctDNA alterations detected. | | | | | | | | | | | | | |  |
| --- | --- | --- | --- | --- | --- | --- | --- | --- | --- | --- | --- | --- | --- | --- |
| Case number | Gender | Age | BMI | Race | Smoking | Stage | # Metastasis site | Metastasis | Survival | Group | Test | On therapy at time of blood draw? | Treatments | |
| 1 | Male | 65-75 | Overweight | White | Never | IV | 1 | Yes | Alive | CUP | G | N | No | |
| 2 | Male | <55 | Overweight | Black | Current/Recent | IV | 0 | No | Alive | CUP | G | N | No | |
| 3 | Female | 55-65 | Underweight | White | Former | III | 1 | Yes | Dead | LUAD | FG | Y | CARBOPLATIN CHEMO INFUSION (BY AUC) FOR PROTOCOLS | |
| 4 | Male | <55 | Normal | White | Former | II | 0 | No | Alive | Headneck | FG | N | No | |
| 5 | Female | <55 | Normal | White | Current/Recent | IV | 2 | Yes | Alive | LUSC | G | N | No | |
| 6 | Male | <55 | Obese | White | Former | IV | 2 | Yes | Alive | LUAD | FG | N | No | |
| 7 | Female | 55-65 | Overweight | Black | Never | IV | 1 | Yes | Alive | CUP | FG | Y | DOXORUBICIN LIPOSOMAL CHEMO INFUSION | |
| 8 | Male | 75-90 | Overweight | White | Former | IV | 1 | Yes | Alive | Headneck | G | N | No | |
| 9 | Male | <55 | Normal | Asian | Former | IV | 0 | No | Alive | Headneck | G | Y | CARBOPLATIN CHEMO INFUSION (BY AUC) FOR PROTOCOLS | |
| 10 | Female | <55 | Normal | OTHER | Never | IV | 0 | No | Alive | Headneck | G | Y | CISPLATIN CHEMO INFUSION (WITH MANNITOL) | |
| 11 | Male | 55-65 | Obese | White | Former | IV | 0 | No | Alive | OtherGI | FG | Y | RAMUCIRUMAB CHEMO INFUSION | |
| 12 | Male | 65-75 | Normal | White | Current/Recent | IV | 1 | Yes | Alive | Pancreas | G | Y | FLUOROURACIL CHEMO INFUSION,  GEMCITABINE CHEMO INFUSION,  INV-CHEMO-CPI-613 INFUSION,  IRINOTECAN CHEMO INFUSION,  OXALIPLATIN CHEMO INFUSION,  PACLITAXEL PROTEIN BOUND CHEMO INFUSION STRAIGHT DRUG | |
| 13 | Male | <55 | Obese | White | Current/Recent | II | 0 | No | Alive | Colorectal | G | Y | BEVACIZUMAB CHEMO INFUSION,  FLUOROURACIL CHEMO INFUSION,  IRINOTECAN CHEMO INFUSION | |
| 14 | Female | 65-75 | Obese | White | Current/Recent | IV | 0 | No | Alive | Colorectal | G | N | No | |
| 15 | Female | <55 | Normal | White | Current/Recent | IV | 0 | No | Dead | LUAD | FG | Y | CARBOPLATIN CHEMO INFUSION (BY AUC) FOR PROTOCOLS | |
| 16 | Female | 55-65 | Obese | White | Former | IV | 0 | No | Alive | Colorectal | FG | N | No | |
| 17 | Female | <55 | Overweight | White | Current/Recent | IV | 1 | Yes | Dead | OtherGI | FG | Y | FLUOROURACIL CHEMO INFUSION,OXALIPLATIN CHEMO INFUSION | |
| 18 | Male | 55-65 | Normal | White | Never | IV | 0 | No | Dead | NSCLC-NOS | G | N | No | |

| Table S2. Description of mutation load in total cohort (N=177). | | | | | | |
| --- | --- | --- | --- | --- | --- | --- |
| **Characteristic** | **Overall survival (Logistic P value)** | **Range of mutation load** | **Median of mutation load** | **Mean of mutation load** | **P value(Wilcoxon)** | **P value(K-W)** |
| **Gender** | 0.175 |  |  |  |  | 0.6051 |
| Male |  | 0-23 | 3 | 3.62 | ref |  |
| Female |  | 0-16 | 2 | 3.47 | 0.6062 |  |
| **Age** | 0.0323* |  |  |  |  | 0.05061* |
| <55 yrs |  | 0-9 | 1.5 | 2.5 | ref |  |
| 55-65 yrs |  | 0-14 | 4 | 4.145 | 0.01* |  |
| 65.1-75 yrs |  | 0-23 | 3 | 3.526 | 0.03986* |  |
| 75.1-90 |  | 0-16 | 2 | 3.778 | 0.1166 |  |
| **BMI** | 0.636 |  |  |  |  | 0.04215* |
| Underweight (<18.5) |  | 0-6 | 1.5 | 1.75 | ref |  |
| Normal (18.5<=BMI<25) |  | 0-23 | 3 | 4.095 | 0.01453* |  |
| Overweight (25<=BMI<30) |  | 0-13 | 2 | 2.982 | 0.1324 |  |
| Obese (>=30) |  | 0-12 | 3.5 | 3.882 | 0.05044* |  |
| **Smoking history** | 0.296 |  |  |  |  | 0.8861 |
| Current/Recent |  | 0-9 | 3 | 3.038 | 0.7467 |  |
| Former |  | 0-14 | 2 | 3.642 | 0.9065 |  |
| Never |  | 0-23 | 3 | 3.932 | ref |  |
| **Race** | 0.943 |  |  |  |  | 0.3091 |
| White or Caucasian |  | 0-23 | 3 | 3.52 | ref |  |
| Black or African American |  | 0-10 | 4 | 3.792 | 0.3102 |  |
| **Stage** | 0.0378* |  |  |  |  |  |
| StageⅠ |  | 1-9 | 3 | 3.846 | ref | 0.6099 |
| StageⅡ |  | 0-7 | 2 | 2.667 | 0.3079 |  |
| StageⅢ |  | 0-14 | 2 | 3.179 | 0.2968 |  |
| StageⅣ |  | 0-23 | 3 | 3.713 | 0.6904 |  |
| **Metastasis** | 0.93 |  |  |  |  | 0.166 |
| Yes |  | 0-14 | 2 | 3.165 | ref |  |
| No |  | 0-23 | 3 | 3.88 | 0.1664 |  |
| **# of Metastasis sites** | 0.25 |  |  |  |  | 0.2142 |
| 0 |  | 0-14 | 2 | 3.165 | 0.02418* |  |
| 1 |  | 0-23 | 3 | 3.672 | 0.05877 |  |
| 2 |  | 0-9 | 3 | 3.333 | 0.1543 |  |
| ≥3 |  | 1-16 | 7 | 6.2 | ref |  |
| **Vital status** |  |  |  |  |  |  |
| Alive |  | 0-23 | 2 | 3.008 | ref |  |
| Dead |  | 0-16 | 4 | 4.593 | 0.00069* |  |

| Table S3. All mutations detected in patient ctDNA compared with other studies. | | | | | | | | | | |
| --- | --- | --- | --- | --- | --- | --- | --- | --- | --- | --- |
| Rank | Wake Forest cohort (N=177) | | Wake Forest lung cancer cohort (N=103) | | *Tompson* et al. cohort (N=102) | | *Schwaederle* et al. cohort (N=171) | | *Villaflor* et al. cohort (N=68) | |
| 1 | TP53 | 47.5 | TP53 | 53.4 | EGFR | 54.9 | TP53 | 29.8 | TP53 | 40 |
| 2 | KRAS | 20.9 | KRAS | 27.2 | TP53 | 47.1 | EGFR | 17.5 | EGFR | 21 |
| 3 | EGFR | 19.2 | EGFR | 23.3 | NF1 | 14.7 | MET | 10.5 | KRAS | 21 |
| 4 | PIK3CA | 15.3 | PIK3CA | 15.5 | APC | 12.7 | PIK3CA | 7 | APC | 11 |
| 5 | ERBB2 | 13.6 | BRCA1 | 15.5 | KRAS | 10.8 | NOTCH1 | 5.8 | AR | 7 |
| 6 | MYC | 13 | ERBB2 | 14.6 | ERBB2 | 9.8 | ALK | 4.7 | MET | 7 |
| 7 | BRCA1 | 11.9 | NF1 | 12.6 | ARID1A | 9.8 | ERBB2 | 4.7 | CDKN2A | 6 |
| 8 | NF1 | 10.2 | MYC | 12.6 | PDGFRA | 9.8 | KRAS | 4.1 | PIK3CA | 6 |
| 9 | MET | 9.6 | KIT | 11.7 | FGFR2 | 7.8 | APC | 2.9 | NOTCH1 | 6 |
| 10 | ARID1A | 9 | PDGFR1 | 10.7 | PIK3CA | 6.9 | AR | 2.9 | ERBB2 | 5 |
