# Supplementary material for: Circulating mutational portrait of cancer: manifestation of aggressive clonal events in both early and late stages
Source: J Hematol Oncol. 2017 May 4;10:100. doi: 10.1186/s13045-017-0468-1 (PMC5418716; doi:10.1186/s13045-017-0468-1)

Additional file 2:

Figure Legends:

Additional file 2: Figure S1. ctDNA mutational landscape for major cancer groups for the top 30 genes in total cohort.

Additional file 2: Figure S2. ctDNA mutational landscape of patients’ stage known for the top 30 genes in lung cancer cohort.

Additional file 2: Figure S3. Higher mutation numbers in the ctDNA are associated with decreased survival in lung cancers. Higher mutation numbers in ctDNA are associated with poor survival. “n” defines the number of mutations and survival plots are separated by mutation numbers: n=1, 2, 3, 4, 5, and 6 mutations. Blue lines indicate more than “n” mutations and the pink lines indicate equal to or less than “n” mutations. P-values were derived using the log-rank test.

Additional file 2: Figure S4. The concordance of top mutated genes in the ctDNA and tDNA of 37 lung cancer patients.

Additional file 2: Figure S1. ctDNA mutational landscape for major cancer groups for the top 30 genes in total cohort.

Additional file 2: Figure S2. ctDNA mutational landscape of patients’ stage known for the top 30 genes in lung cancer cohort.

Additional file 2: Figure S3. Higher mutation numbers in the ctDNA is associated with decreased survival in lung cancers.

Additional file 2: Figure S4. The concordance of top mutated genes in the ctDNA and tDNA of 37 lung cancer patients.


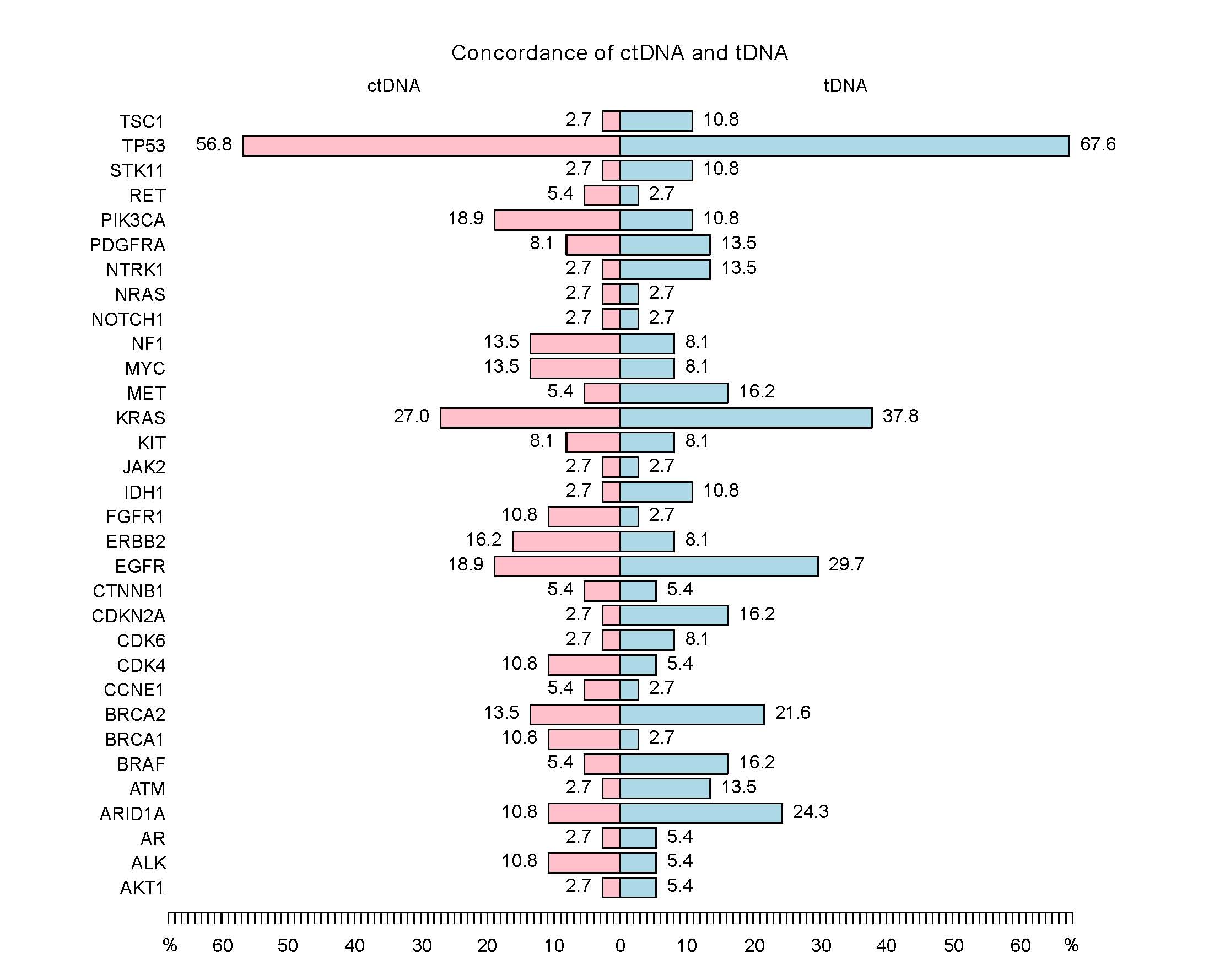

Supplement: Supplementary file 2 — ctDNA mutational landscape for major cancer groups for the top 30 genes in total cohort. Figure S2. ctDNA mutational landscape of patients’ stage known for the top 30 genes in lung cancer cohort. Figure S3. Higher mutation numbers in the ctDNA are associated with decreased survival in lung cancers. Higher mutation numbers in ctDNA are associated with poor survival. “n” defines the number of mutations and survival plots are separated by mutation numbers: n = 1, 2, 3, 4, 5, and 6 mutations. Blue lines indicate more than “n” mutations and the pink lines indicate equal to or less than “n” mutations. P-values were derived using the log-rank test. Figure S4. The concordance of top mutated genes in the ctDNA and tDNA of 37 lung cancer patients. (DOC 1098 kb) [file 13045_2017_468_MOESM2_ESM.doc]
